# Pharmacopœial Doses

**Published:** 1906-05-19

**Authors:** 


### PHARMACOPŒIAL DOSES.

The structure and constitution of the national Pharmacopœia is a question to which the medical profession cannot possibly remain indifferent. It is true that the substances named in that volume in no sense limit the range of the prescriber's activity, and that non-official, equally with official remedies are open to his selection and choice. All the same, it must be recognised that pharmacopœial rank does establish in the mind of the practitioner, and especially of the junior practitioner, a certain favourable prepossession, the influence of which can hardly be questioned. This, on the whole, is a healthy position, and is certainly to be preferred to one created by more or less irresponsible advisers and by the persevering and ingenious efforts of commercial organisations. Though, in our judgment at least, there ought not to be any pretence that the compilers of the Pharmacopœia are competent in any special measure to appraise the therapeutic value of remedies and to attach to those which happen to find favour in their sight a conclusive hall-mark or guarantee, it still remains true that to these authorities is accessible a mass of testimony of the extent to which individual remedies are used and trusted by the profession far beyond what can possibly be in the possession of the private practitioner. Hence, without doubt, remedies recognised by the Pharmacopœia occupy a somewhat special position, and present what may be called a claim for preferential attention; and, provided that the qualifications above alluded to are admitted, this arrangement possesses a balance of advantage.

The Pharmacopœia, however, not only names and describes various remedies, but also, in the case of those used for internal administration, suggests the doses in which they are, or may be, employed. Whether this is wise or unwise seems a fit subject for discussion, and the whole question has recently been debated at one of the metropolitan medical societies. Certainly it is important to secure in the profession an accurate estimate of the main purpose and value of the official doses, for upon this point there appears to be a somewhat widespread misapprehension. In the preface to the official volume it is carefully pointed out that the doses stated in the volume "are meant for general guidance, but are not authoritatively enjoined by the Council." Considering that the Council has no power to "enjoin" doses this disclaimer has a decidedly gratuitous quality, but, all the same, is of importance in reference to the principle which it asserts. That principle finds expression in the sentence, "the medical practitioner must act on his own responsibility as to the doses of any therapeutic agents which he may administer." From this it is manifest that the statement of doses in the Pharmacopœia is not intended either to decide the judgment of the practitioner or to limit his freedom of action. Such a condition is indeed essential to sound medical practice, which demands a personal knowledge of all the circumstances in each individual case, and liberty to act as these seem to direct. The suggestion that some corporate authority has the right or ability to control or limit the free judgment and course of action of the practitioner is entirely opposed both to the traditions of the profession and to the interests of the public. Direct and immediate personal responsibility, as opposed to the shadow and support of tradition and rule, is the very atmosphere of medical life and work. All this, so far as the administration of remedies is concerned, is fully recognised in the sentences already quoted, from which it is evident that the Pharmacopœia makes no pretence to invade the area of the practitioner's responsibility. But whilst the Pharmacopœial doses are transferred bodily to text-books and lectures, the qualifying phrases of the "Preface" generally fail to receive an equal compliment. Hence, it cannot be doubted, these doses come to the medical student with a voice and claim which those who have framed them expressly disclaim. As a result they receive an artificial and unwarranted sanction, and are apt to gain an influence in the life of the practitioner much in excess of their meaning. In this way the maximum official dose comes to act as a limit and barrier, beyond which it is not safe to pass. As opposed to this is the therapeutic canon that successful treatment needs an estimate and experience of the wants and peculiarities of the patient immediately concerned, and an adjustment alike of remedy and dose to the specific claims of the individual case. Prescribing by rule and red tape calls for energetic discouragement. And, as an undue deference to the doses quoted in the Pharmacopœia is an influence tending to promote such a practice, it is well that these doses should be shorn of any sanctity suggested by the accident of their position, and that they should be presented in their true meaning and value. This position is readily defined. The principal end to be attained by the introduction of doses into the Pharmacopœia is the protection of the public. They offer an opportunity to the dispenser to approach the physician in the case of any accidental slip or error, and thus diminish the risk of a more or less serious mischance. Regarded in this light, the present arrangement has an abundant defence, and so explained the official doses sink to their true position. They are thus seen to offer no restriction to the decision of the practitioner and no retreat for a defective judgment.

